# Insights into the Pharmacological Effects of Flavonoids: The Systematic Review of Computer Modeling

**DOI:** 10.3390/ijms23116023

**Published:** 2022-05-27

**Authors:** Amir Taldaev, Roman Terekhov, Ilya Nikitin, Anastasiya Zhevlakova, Irina Selivanova

**Affiliations:** 1 Laboratoty of Nanobiotechnology, Institute of Biomedical Chemistry, Pogodinskaya Str. 10/8, 119121 Moscow, Russia; 2 Department of Chemistry, Sechenov First Moscow State Medical University (Sechenov University), 119991 Moscow, Russia; terekhov_r_p@staff.sechenov.ru (R.T.); nikitin_i_d@student.sechenov.ru (I.N.); zhevlakova_a_k@staff.sechenov.ru (A.Z.); selivanova_i_a@staff.sechenov.ru (I.S.)

**Keywords:** computer modeling, molecular modeling, cheminformatics, in silico, docking, flavonoids, phytomedicine, systematic review, limitations, bias risk

## Abstract

Computer modeling is a method that is widely used in scientific investigations to predict the biological activity, toxicity, pharmacokinetics, and synthesis strategy of compounds based on the structure of the molecule. This work is a systematic review of articles performed in accordance with the recommendations of PRISMA and contains information on computer modeling of the interaction of classical flavonoids with different biological targets. The review of used computational approaches is presented. Furthermore, the affinities of flavonoids to different targets that are associated with the infection, cardiovascular, and oncological diseases are discussed. Additionally, the methodology of bias risks in molecular docking research based on principles of evidentiary medicine was suggested and discussed. Based on this data, the most active groups of flavonoids and lead compounds for different targets were determined. It was concluded that flavonoids are a promising object for drug development and further research of pharmacology by in vitro, ex vivo, and in vivo models is required.

## 1. Introduction

Computer modeling is a rapidly developing scientific method that allows us to predict the courses and results of various processes by the use of modern intelligent technologies [1,2]. Molecular modeling is one of the areas of computer modeling, and it is widely used in drug design [3,4,5]. Several methods such as quantitative structure-activity relationship (QSAR) and quantitative structure-property relationship (QSPR) [6,7], molecular docking [8], and molecular dynamics simulations [9,10,11] give an opportunity to identify the potential biologically active compounds and establish the mechanisms of target-ligand interaction. Molecular modeling is used for the prediction of chemical reactivity and synthesis strategy optimization by, for example, density functional theory (DFT) calculations [12,13]. Moreover, this method has several benefits in ADMET prediction for small molecules that have potential as drugs [14]. Of course, the successfully completed computer-performed experiment (in silico) cannot guarantee the future implementation of a biologically active molecule in clinical practice [15,16]. Nevertheless, using molecular modeling, it is possible to identify the potential area of compounds’ medical applications and to assess the necessity of further in vitro and in vivo assays [17,18].

**Figure 1:**
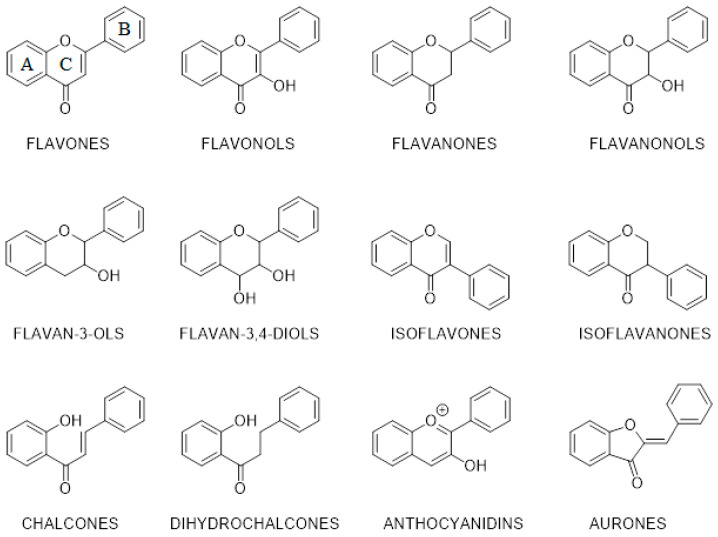
Base structures of classical flavonoid groups.

Flavonoids are widely occurring secondary metabolites of plants. Currently, over 8000 compounds are classified as flavonoids [19]. Quercetin is a textbook example of flavonols that decrease total sick days and severity of upper respiratory tract infection in randomized, double-blinded, placebo-controlled trials [20]. Flavanonol, which belongs to the structural type of quercetin, named taxifolin, demonstrated wound-healing activity [21]. Luteolin, apigenin, and wogonin, which can be classified as flavones, show induced neutrophil apoptosis and have potential as neutrophil apoptosis-inducing anti-inflammatory, proresolution agents [22]. Flavanone hesperetin, in combination with sodium cyclic lysophosphatidic acid, showed significant antiaging effects on skin hydration and elasticity in a single-center clinical trial [23]. In general, data from several studies suggest that higher dietary intake of flavonoids may be associated with better cognitive health [24,25], improved prognosis for cardiovascular diseases [26,27], and decreased body weight [28]. Taken together, due to antioxidant activity, selective affinity to several biological targets, and high safety profiles, flavonoids are a prospective class of natural compounds for drug development [29,30,31,32]. Flavonoids are usually divided into 12 groups based on the structure of the molecule (Figure 1).

The aim of this study is to identify the trends of in silico methods applied as a primary screening tool for drug design on a flavonoid basis.

## 2. Methods

### 2.1. Search Strategy

The systematic review adhered to PRISMA (Preferred Reporting Items for Systematic Reviews and Meta-Analysis) [33] guidelines, including search strategy, selection criteria, data extraction, and data analysis. The papers included in this analysis were selected from the Google Scholar database. Studies were restricted to those published from January 2016 to October 2021 to ensure the literature was relevant. The following keywords were used in the search strategy: “flavonoid”, “molecular modeling”, “chemoinformatics”, “computational chemistry”, “computer modeling”, “docking”, “molecular dynamics”. An example search strategy as applied to the scientific database is shown below in English: (“in silico” OR “molecular modeling” OR cheminformatics OR “computational chemistry” OR “molecular simulation” OR “computer simulation” OR docking OR “molecular dynamics”) AND flavonoid; and in Russian: (“мoлекулярнoе мoделирoвание” OR хемoинфoрматика OR дoкинг OR “мoлекулярная динамика”) AND флавoнoид. Only studies published in the English and the Russian languages were considered for inclusion due to a lack of translation resources.

### 2.2. Review Protocol and Data Extraction

Two independent review authors (I.N. and A.T.) conducted the literature search in the specified scientific databases. Results of the search were collected in Google Drive. Duplicate publications were excluded. The same review authors independently screened publications using the criteria for inclusion (Table 1). 

**Figure 2:**
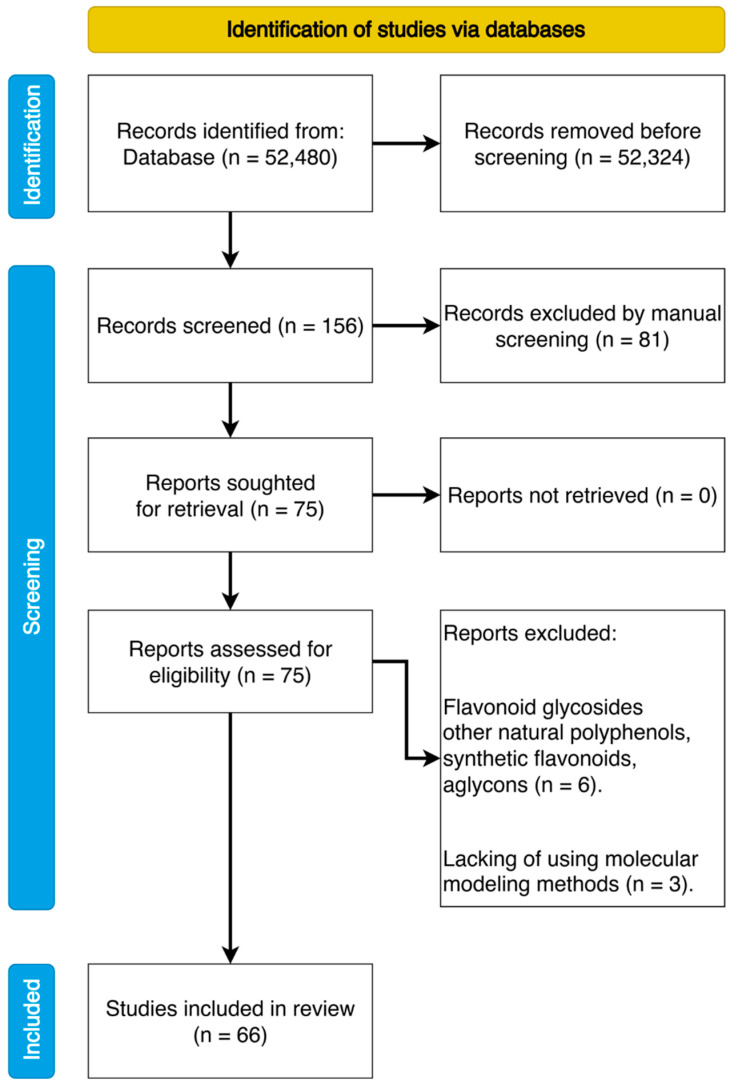
PRISMA flowchart of the search and selection process of the articles.

Resolution of any disagreements occurred through discussion and required consultation with the three other authors (R.T., A.Z., and I.S.) for consensus. As a result of the annotations analysis, 66 articles were selected for in-depth study that met the objectives of this research (Figure 2). Qualitative and quantitative content analysis was performed. Data synthesis and required statistical analyses were conducted by I.N. and A.T. Findings are presented as a narrative synthesis, as well as tables and figures.

### 2.3. Assessment of Risk of Bias

Two review authors (R.T. and A.Z.) made systematic and independent assessments of the risk of bias in each research using the methodological domains presented in Table 2.

Disagreements in judgments about the risk of bias were resolved by discussion or, when necessary, arbitrated by an independent third review author (I.S.).

## 3. Results and Discussion 

### 3.1. General View on Articles Sample

**Figure 3:**
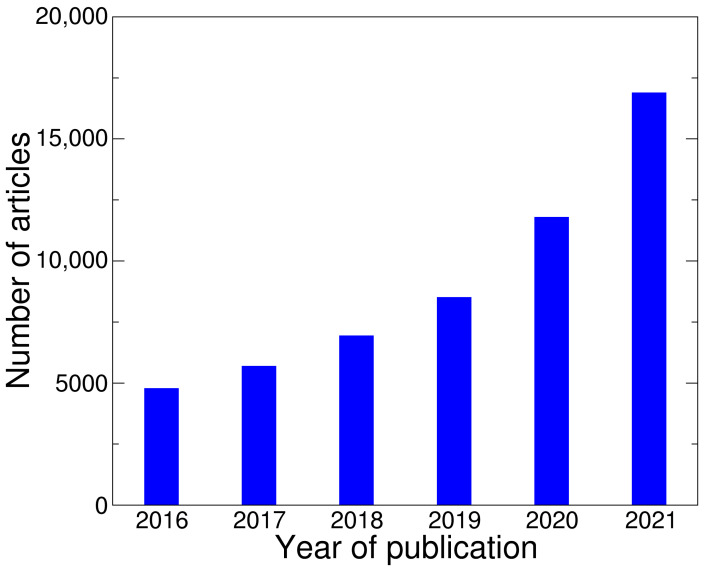
Monitoring of scientific information on molecular modeling of flavonoids (Google Scholar data).

An increasing number of publications on molecular modeling with flavonoids was observed. For example, in 2016, the 4790 articles were published, while in 2021, this parameter was approximately four times higher and was 16,900 (Figure 3).

### 3.2. Molecular Modeling Methods

Specialists in the field of medicinal chemistry show great interest in computer modeling of flavonoid pharmacological activities, which is confirmed by the annual increase of publications on this topic. 

Molecular docking has the greatest prevalence, which was used in 92.4% of the analyzed articles [34,35,36,37,38,39]. Molecular dynamics simulations were revealed in 31.8% of the papers [40,41,42,43]. Pharmacophore modeling, QSAR/QSPR models, quantum chemical, and hybrid quantum mechanical/molecular mechanical methods were found in 21.1% of publications [44,45,46,47,48].

Interestingly, some articles presented the research that was realized in translational design, including experiments in vitro, ex vivo, and in vivo. For example, Sun et al. confirmed the affinity of taxifolin to 11β-hydroxysteroid dehydrogenase 1 in rats and using human HSD11B1, with IC50 values of 37,833 and 4981 nM, respectively [49]. During another translational study, it was shown that taxifolin inhibits aryl hydrocarbon receptors and some proteins of the cytochrome P450 system that may be promising for breast cancer treatment [50]. The publication of such articles is a rather remarkable outcome.

### 3.3. Software and Databases for Molecular Docking

Considering that molecular docking is based on protein-ligand interaction, it was interesting to analyze the software needed to create virtual supramolecular structures. Based on the frequency data of different compactional methods, it was suggested that the majority of researchers prefer to optimize the study design by following the criteria:

- User-friendly software;
- Intuitive graphical interface;
- Lack of need for high-performance computing resources;
- Good predictive ability.

The majority (89%) of target protein structures were obtained from the RCSB Protein Data Bank (PDB) [51,52,53]. In cases when the compounds of interest were not contained in the database (11%), homology modeling was used to construct the necessary structure [45,54,55,56]. 

Three-dimensional structures of ligands were obtained in two ways: by extracting information from various libraries (65%) [57,58,59,60,61] or by constructing a molecule using special software (35%) [62,63,64,65,66]. PubChem (62%) was the most frequently used database, while ZINC (15.0%) and ChemSpider (12.0%) were less common. PubChem belongs to the US National Library of Medicine, and its leadership, despite the smaller number of compounds (about 109 million) compared to ZINC (230 million), is due to having the richest information about each molecule. ChemSpider has a smaller amount of information on each compound and a smaller number of records (about 101 million). Ligand constructors are used only when the molecular structure is not available in the computer library as the most time-consuming method of virtual model generation. The most common software for flavonoid molecular docking is AutoDock (47%), Glide (23%), and GOLD (9%).

### 3.4. Structure—Biological Activity Relationship: Qualitative Analysis

The parent structure of flavonoids is 1,3-diphenylpropane, and the aromatic fragments are designated as ring A and ring B [67]. The majority of flavonoid groups are characterized by the heterocycle (ring C) containing oxygen. This ring may be aromatic (flavones, flavonols, etc.) or not (flavanones, flavanonols, etc.). As the rule, carbonyl and several hydroxyl functional groups are present in the molecular structure of flavonoids that can act as a pharmacophores. 

**Figure 4:**
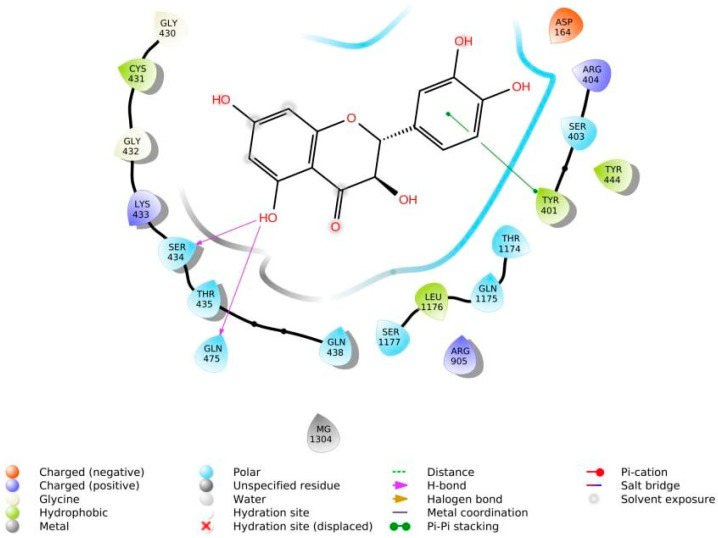
Interaction of taxifolin and P-glycoprotein.

The phenolic hydroxyl groups of the studied natural compounds serve as H-bond donors. In cases when the hydrophobic interactions play a key role, the presence of the methoxy group leads to an increase of affinity to the target compared with the hydroxyl group [68]. Due to aromatic rings, the π,π-interactions with the side residues of heterocyclic and aromatic α-amino acids (tryptophan, histidine, phenylalanine, and tyrosine) are possible [69]. Figure 4 demonstrates all types of interactions.

It was found that the antiangiogenic potential of the flavonoid depends on the presence of a C2-C3 double bond [70]; the hydroxyl group in the position 3′ of the ring C contributes to an increase in antioxidant, anti-inflammatory, and antitumor activity [71]. If, along with the multiple C2-C3 bonds, a catechol group is present in the ring B, then such a molecule demonstrates a high affinity for the angiotensin-converting enzyme [72]. Substituents 3-OH, 5-OH, 6-OMe, 6-OH, 7-OH, 3′-OH, and 4′-OMe were identified as key fragments of the molecules when interacting with multidrug resistance-associated protein 2 (MRP2) [55]. 

It was also interesting to determine the specificity of the interaction of flavonoid groups. Thus, flavones (6-hydroxyluteolin, scutellarein), flavonols (kaempferol), and flavanones (naringenin, eridioctyol) exhibit a high affinity to the estrogen receptor α (ERα), which has been proven in both AutoDock and Glide software. Representatives of these groups of flavonoids can be recommended in the development of antitumor drugs for the treatment of breast cancer [71,73]. Interaction with this protein target results in several types of patient management, such as estrogen hormone replacement therapy and preventive care for breast cancer [74]. Flavones (baicalein, ladanein), flavonols (quercetin), and their glycosylated forms (baicalin) interact with the E protein of various strains of the dengue virus causing fever with a similar name [54]. Such ligands may be used in the treatment of this disease [75]. It is worth noting that the width of the confidence interval of the scoring function calculated for flavones is quite large. This indicates a different degree of protein-ligand binding within this group. Flavones (5-hydroxyflavone) and flavonols (quercetin) have a high affinity for the potassium channel Kir6.1, acting on which some cardiovascular diseases can be treated [56]. Flavones (luteolin, apigenin) can serve as the basis of drugs that control the pathogenicity of *Helicobacter pylori* due to their ability to bind to one of the main virulence factors of bacteria of this species—vacuolating cytotoxin protein (VacA) [76]. Flavonols (quercetin), their glycosides (avicularin, hyperoside), and flavanonols (taxifolin) with comparable effects function as arginase inhibitors, which is a potential target for the development of new approaches to the treatment of leishmaniasis [77]. Flavan-3-ols (catechin, epicatechin) are characterized by the best values of the scoring function when binding to the CA II-F complex in comparison with flavones, flavanones, and flavanonols and are of interest for the treatment of fluorosis [78]. According to the silico results, flavanones (eriodictyol) and flavanonols (taxifolin) are able to inhibit transcription factors Tec1 and Rfg1 because they can be used in the treatment of infection caused by *Candida albicans* fungus [79].

### 3.5. Structure—Biological Activity Relationship: Quantitative Analysis

Meta-analyses of scoring functions calculated during molecular docking was studied in [54,56,71,73,76,77,78,79]. General information about the average affinities of each flavonoid group to the biological targets is presented in Table 3 and Table 4 for AutoDock and Glide software, respectively.

Table 3 provides the experimental data on docking flavonoids of different groups to several biological targets, as performed by AutoDock. The extracted affinity values demonstrate the potential ability of small molecules to form supramolecular complexes with selected proteins in their active sites resulting in multiple pharmacological effects. Apparently, flavanonols form significantly more stable complexes with ERα compared with flavonols, which was approved by in vitro experiment [57]. This may be explained by the non-plane structure of the carbon skeleton, which can be quite similar to estrogen. Furthermore, the affinity value for flavones with protein VacA seems high compared to other targets, so it may be interesting to perform similar calculations for other flavonoid groups.

The results obtained from the meta-analysis of affinity values of flavonoid groups to target proteins calculated via Glide are summarized in Table 4. Compared with the previous table, the heterogeneity of values is obvious. The scoring functions for flavonoids docking with complex CA II-F and RFG1 were notably lower than for other biological targets. Surprisingly, the results of Glide calculations confirmed the previous in silico data obtained by AutoDock: Flavonones demonstrate a significantly higher affinity with ERα and RFG1 than other flavonoid groups. At the same time, flavan-3-ols show significantly higher scoring functions with complex CA II-F. 

In general, the data of Glide calculations seemed more appropriate for decision support in further study design due to significant differences in affinity values compared with AutoDock.

### 3.6. Limitation Section

The study was performed in agreement with the PRISMA guidelines to prevent reviewer biases in the review process, including independent, duplicate inclusion and exclusion of identified studies, risk of bias assessments, and data extraction.

Nevertheless, it is important to notice that systematic reviews summarize the limitations of primary studies [80]. Thus, to measure the reliability of current research, it was necessary to assess the bias risk in the studies included in the review. There are many guidelines for considering bias in papers for randomized controlled clinical trials. However, to our data, there is no such instrument for in silico studies. Because of this, we developed average criteria that can serve this goal based on the literature information and considered discussion with professional society (see Table 2).

The domains included in our research primarily cover all types of bias that are currently understood to affect the results of molecular docking. These are:

- Bias arising from ligands selection process;
- Bias due to ligands structure optimization;
- Bias arising from protein target selection process;
- Bias due to protein target processing;
- Bias arising from molecular docking process;
- Bias in results assessment.

Ligand filtering results in optimization of calculation processes and gives opportunity to exclude the molecules with huge volumes, low safety profiles, and inappropriate pharmacokinetic behavior from the ligand set [81,82]. Ionization is a critical parameter of ligand structure because it impacts the formation of a supramolecular complex with the target. Because of this, the ionization assessment should be performed correctly with reference to the p*K_a_* value of small molecules and the pH value of media [83,84]. Another important stage of ligand structure optimization is the generation of all possible conformers that would be characterized by the lowest potential energy and optimal bond lengths, angles, and dihedrals. The affinity of conformers may differ significantly, so it influences the bias risks [85,86]. The resolution is one of the most obvious characteristics of the protein target models. In case it is higher than 2.5 Å, the position of atoms cannot be identified unequivocally, and such structures should not be used for molecular docking [87]. Furthermore, the method of target structure generation makes sense because only the NMR spectroscopy gives an opportunity to get the 3D model at the conditions that would be near to the biological ones [88]. However, it was shown that the protonation of amino acid residues, as well as the addition of missing residues and side chains, that was performed by a special toolkit, decreased the bias risks during the target structure generation by X-ray crystallography and cryogenic electron microscopy [89,90]. Checking the histidine charge and addition of metals are the critical stages that should be performed during the protein target processing to make the model biochemically correct [91,92]. The choice of an appropriate tool for molecular docking is a main issue of the bias domain. The software differs by using algorithm methods and scoring functions. The Monte-Carlo algorithm and empirical scoring functions demonstrate better results compared with other calculation methods and are confirmed by in vitro methods [87,93]. Finally, to assess the results of molecular docking correctly, we should be sure that the parameters of modeling are appropriate for the analyzed system, and the re-docking serves this goal [94]. The visual control of docking results is an essential part of in silico research that helps exclude the presence of structural artifacts that are formed by computer calculation [95]. Of course, the verification of the molecular docking results by in vitro study should be performed. Only chemical experiments may confirm the results of theoretical modeling [96]. Additionally, the risk of bias by a conflict of interest of authors was assessed using the information regarding funding source. 

**Figure 5:**
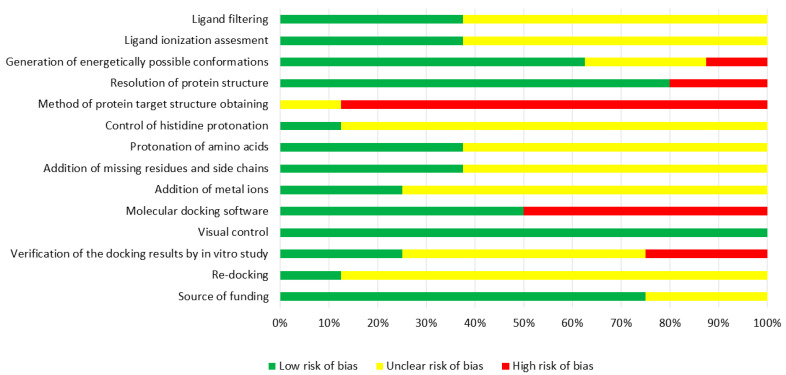
Risk of bias graph.

The developed methodology was implemented in the articles (Figure 5) and used for the meta-analysis [54,56,71,73,76,77,78,79]. Whilst this study included a total of eight papers, there is very poor information about the processing of protein targets and the assessment of results of molecular docking. Apparently, it is a result of a gap in clear guidelines of reporting for in silico studies that would sign the critical issues of such research. Moreover, half of the studies or even more reported the use of methods during protein target selection and molecular docking characterized by a confirmed high risk of bias. It may be associated with the absence of suitable target models and free software. Based on the data, it can be considered that there are limitations in the applicability of the evidence. Of course, these results of bias risk assessment in our meta-analysis cannot be transferred to all studies that conducted the molecular docking of flavonoids. However, the tendency reflects the necessity of paying more attention to the quality of such papers. Nevertheless, the conflict of interests was not identified in the analyzed studies.

We could not assess publication bias with a Begg funnel plot or an Egger test because the included studies were quite heterogeneous. In general, we may have missed some potentially eligible studies in languages other than English or Russian and studies with negative results.

We have not identified any other systematic reviews or meta-analyses that evaluated the pharmacological effects of flavonoids based on in silico studies. Meanwhile, we cannot draw any conclusions about agreements and disagreements with other reviews.

### 3.7. Lead Compounds

**Figure 6:**
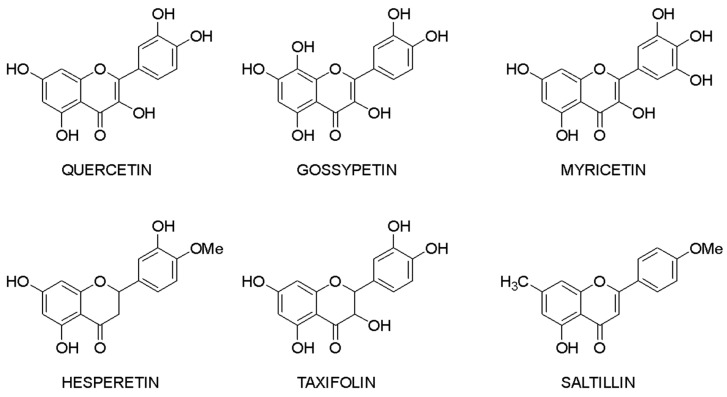
Lead compounds.

Molecular docking makes it possible to evaluate the affinity of the ligand with the target. Based on this criterion, it is possible to compile a list of the most active compounds for each specific interaction of a flavonoid with a macromolecule. The structures of the leader compounds are shown in Figure 6. Thus, taxifolin (dihydroquercetin) has a high potential for tuberculosis therapy, as it has demonstrated the ability to interact with DNA gyrase and aminoacyl-tRNA synthetase—two enzymes involved in the translation, transcription, and replication of bacterial DNA [97]. The pathogenic effect of the Ebola virus can be disrupted by affecting the vital protein structures VP40, VP35, VP30, and VP24 with flavonoids such as gossypetin or taxifolin. These compounds proved to be leaders in the study of more than 4500 flavonoids [98]. It is interesting to notice that despite the near structures of the molecules, they have similar interaction patterns with only two proteins among four targets. Both taxifolin and gossypetin can form H-bonds with His124, Gly126, and Gln170 in VP40. Furthermore, they interact with Gln103, Ser123, Asp124, and Asn185 or Gln184 in the active site of VP24. Key amino acid residues in VP35 are Gln244(2) and Asp302 for taxifolin, while for gossypetin, it is only the Gln241(2). Finally, gossypetin interacts with Asp158(2), Arg196, and Gln233(2) in VP30. However, taxifolin forms H-bonds with Arg196, Gly200(3), Gln233, Ser234, and Phe238 residues of the VP30. Differences in the affinity of these flavonoids are apparently associated with the spatial structure of the heterocyclic ring. Gossypetin has a plane aromatic structure, and the taxifolin molecule is characterized by two chiral centers. Thus, taxifolin can exist as four stereoisomers. Saltillin, taxifolin, and 6-methoxyflavone have a high affinity for *N*-myristoyltransferase (NMT), a target for the treatment of candidiasis [99]. There is also information indicating the feasibility of studying a number of flavonoids in the following diseases: lung cancer [100], breast cancer [50,71], metabolic syndrome [49], pathological conditions caused by *Pseudomonas aeruginosa* [101], hypoestrogenism [102], and depressive disorders [103]. 

Taxifolin is one of the most promising lead compounds, mentioned in Figure 6, due to its wide range of pharmacological activity, rich raw material base, and high safety profile. Prospective taxifolin use in medicine was discussed in a number of articles and reviews [104,105,106]. Moreover, in Russia, this compound is registered as an active pharmaceutical ingredient, and in Europe, it is a supplement (Regulation EU 2017/2470). (2*R*,3*R*)-taxifolin is the most investigated isomer.

It is known that the (2*R*,3*R*)-taxifolin obtained from natural sources is safe [107]. The low toxic potential of taxifolin was predicted by different methods, such as the ORISIS DataWarrior program [108]. Furthermore, it is quite important to note that not only the major *trans*-stereoisomer is characterized by a high safety profile. The VirtualToxLab platform determined (2*S*,3*S*)-taxifolin as one of the lowest toxic molecules from the set of 29 molecules that demonstrated affinity to the SARS-CoV-2 main protease [109]. This software is based on calculating individual binding affinities to 16 validated off-targets, including intracellular receptors, metabolic enzymes, and the hERG potassium channel. Taxifolin is the safest flavonoid in comparison with eriodictyol, luteolin, isoscutellarein, and quercetin. It has shown in silico potential as the main protease of SARS-CoV-2 [108].

Despite the great pharmacological potential, this flavonoid is characterized by low bioavailability [110,111]. To explain the observed efficacy, research on taxifolin metabolism was performed. More than 190 structures of different compounds were found as the result of taxifolin biotransformation by the HPLC-MS/MS method [112]. There was research that tested most of them as COX-2 inhibitors by in silico study [39]. To evaluate the affinity of metabolites to target, molecular docking was performed. A significant number of these compounds were absent in databases. For this reason, the ligand set of 214 3D-structures of all taxifolin stereoisomers and their metabolites was generated manually using ChemBioDraw Ultra (v. 13.0, PerkinElmer, Waltham, MA, USA). Then, the GOLD software (v. 5.4, CCDC, Cambridge, UK) was used to conduct the modeling of intramolecular interaction. During this research, all metabolites were classified into three groups. Compounds that contain all three rings of the initial structure (A, B, and C—see Figure 1) can interact with the three most important amino acid residues in the active site of the target: SER353, SER530, and ARG513. Metabolites that contain two rings (A, C or B, C) do not interact with ARG513 and, for this reason, are less selective. The third group consists of molecules with only one ring. These compounds have a low affinity to COX-2. Thus, research in the field of the design of anti-inflammatory drugs based on taxifolin should be continued.

**Figure 7:**
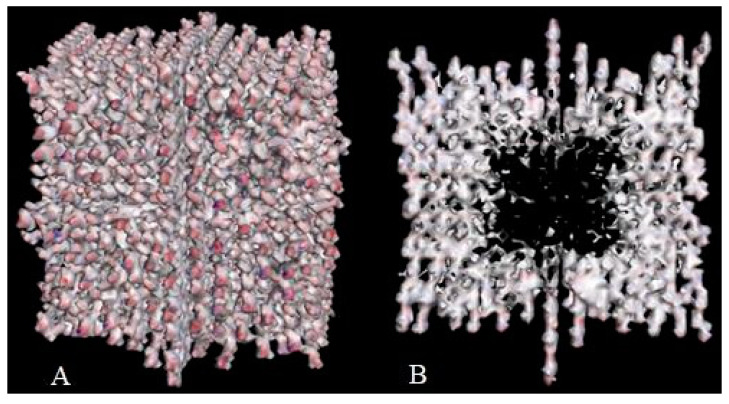
The view of taxifolin nanoparticle (**A**) and its cross-section (**B**) [113].

Crystal engineering is another area of taxifolin research. Microtubes are one of the most significant achievements in the development of new crystal forms in the last 5 years [113]. They were synthesized by precipitation with water from an ethanol solution of taxifolin in the presence of urea. This modification showed notable differences in comparison with the initial active pharmaceutical ingredient. To characterize the properties of the new taxifolin forms in silico, the 3D-models of nanoparticles were generated based on the different crystal unit structures. The simulation of the nanoparticle deformation was carried out by molecular dynamics modeling to evaluate the physical characteristics of taxifolin modifications. This investigation was performed using Materials Science Suite 2018-2 (Schrodinger, New York, NY, USA). A cross-shaped pore was observed at the core of the taxifolin nanoparticle (Figure 7). The diameter of this pore varied from 4 nm at the narrow part to 11 nm at the widest part. These findings make it possible to suggest that the taxifolin microtubes are a mesoporous material. Taxifolin microtubes may have an application in drug delivery.

A wide range of pharmacological activity of flavonoids is a potential for the design of drugs on these bases. After the in silico stage, preclinical and clinical trials are required to verify the safety and efficacy of the candidate molecule. At this moment, on the website https://clinicaltrials.gov (accessed on 25 January 2021) 253 clinical trials of drugs containing flavonoids have been registered. It is worth noting that the number of trials currently being conducted or planned for the near future is 45, and these include pathologies such as post-thrombotic syndrome, sickle cell anemia, chronic kidney disease, psoriasis, glaucoma, type 2 diabetes, and others. It is interesting to note that the possibility of flavonoids being used for the COVID-19 treatment is being studied in 11 trials.

### 3.8. COVID-19 Treatment 

Undoubtedly, the Coronavirus disease 2019 (COVID-19) pandemic is one of the most socially important problems nowadays. There have been approximately 448 million confirmed cases of COVID-19, including more than 6 million deaths, reported by the World Health Organization (WHO) [114]. According to these epidemiological data, it is extremely important to find effective remedies against COVID-19. The flavonoid group is a promising object for this purpose. Indeed, thousands of investigations have been carried out. Obviously, most of them were conducted by in silico approaches. 

For instance, one study aimed to establish flavonoid’s affinity to the spike protein of SARS-CoV-2. The ligand set included apigenin, chrysin, fisetin, galangin, hesperetin, luteolin, morin, naringin, quercetin, and rutin. Docking studies showed that all flavonoids demonstrate considerably high binding affinity, but naringin is characterized by the highest one. This compound shared hydrophobic interactions with the following residues: Asn290, Ile291, His374, Leu370, Leu410, Ala413, Pro415, Phe438, and Gln442. Furthermore, hydrogen bonds were formed with Asp367, Thr371, Lys441, Glu406, and Ser409 amino acid residues [115]. 

Six polyphenolic compounds, namely leucopelargonidin, morin, myricetin, eriodictyol, taxifolin, and enterodiol, exhibited a significant binding affinity for SARS-CoV-2 papain-like protease (PLpro) and main protease (Mpro) during another investigation via molecular docking [116]. The analysis of free binding energy showed that taxifolin has the highest affinity against Mpro and forms H-bonds with Cys145, Ser144, Gly143, Asn142, Leu141 (hydroxyl groups of ring A), Glu166, Met165, His164 (carbonyl group of ring C), Tyr54, Pro52, and Met49. Morin was determined as a compound with the highest affinity toward Plpro. Hydrogen bonding interactions of morin were observed with the following amino acid residues: Gly266, Asn267 (hydroxyl groups of rings A and B), Thr301 (carbonyl group of ring C), Tyr273, Tyr264, and Tyr268 (phenolic hydroxyl groups of ring B). Moreover, further molecular dynamics simulation showed that the binding of bioactive molecules on the corresponding proteases is characterized by structural changes that can disrupt the functions of enzymes and, thus, enhance their antiviral activity. 

Another approach considers angiotensin-converting enzyme 2 (ACE-2) receptors as a target. Molecular docking was used to predict the activity of flavonoid sets, including hesperetin, chrysin, kaempferol, galangyn, myricetin, and rutin [117]. The last molecule showed the best binding affinity to the ACE-2 enzyme. It was found that rutin forms the strongest hydrogen bond with Asn149, Met270, His345, Lys363, Asp368, and Thr445 amino acid residues of the ACE-2 protein. Likewise, this compound has the π-anion interaction with Arg269, π-π stacking interaction with Phe274, π-alkyl interaction with Ala153, and alkyl interaction with Pro346, Met360, and Cys361 residues.

Moreover, in our recent study, 163 flavonoids were screened [118]. ATP-binding domain SP3, main protease NSP 5, RNA-dependent RNA polymerase NCP12, and endoribonuclease NCP15 were considered as targets. Much of the recent COVID-19 treatment research in silico has focused on the identification of lead compounds, while our study pays more attention to the binding affinity of the all-flavonoids groups to biological targets. The median binding energies were −7.4, −7.4, −8.9, and −7.3 kcal/mol for the ADP-binding domain NSP3, main protease NSP5, RNA-dependent RNA polymerase NSP12, and endoribonuclease NSP15, respectively. Therefore, these data suggest that flavonoids can find application in the complex therapy of COVID-19.

The potential benefits of flavonoids in COVID-19 therapy were confirmed by a randomized controlled trial [119]. Groups that obtained quercetin in combination with antiviral drugs demonstrated significantly lower serum levels of critical markers involved in COVID-19 severity and better respiratory rate. However, further clinical trials with other flavonoids and bigger group sizes are required.

These results are very promising and require further research.

## 4. Conclusions

Currently, there is a trend of article numbers increasing, which focuses on the computer modeling of flavonoid interactions with biological targets. Such studies help to accumulate the data on lead compounds that can find medicinal implementation, including COVID-19. In drug discovery, in silico research plays the role of the first screening stage. Assessment of bias risk showed the necessity of clear guidelines of reporting for in silico studies that would sign the critical issues for such research. The results of computational calculations should be supported by a large-scale study of candidate molecules by in vitro, ex vivo, and in vivo methods. The implementation of this translational research model is a promising way for the creation of new phytomedicines based on flavonoids.

## Figures and Tables

**Table 1:** Overall inclusion and exclusion criteria for publication screening.

Section	Criteria	Include If:
Language	English	Yes
	Russian	Yes
Design	In silico studies, complex translational studies with the molecular modeling stage	Yes
	In vitro and in vivo studies, reviews, editorials, letter to the editor	No
Content	Studies examining the affinity of natural flavonoids aglycons to different biological targets	Yes
	Studies examining the affinity of synthetic flavonoids aglycons to different biological targets	No
	Studies examining the affinity of flavonoids glycosides or other natural polyphenols to different biological targets	No
Access	Full-text article accessible	Yes

**Table 2:** Overall inclusion and exclusion criteria for publication screening.

Bias Domain	Issue	Low Risk of Bias	High Risk of Bias	Unclear Risk of Bias
Ligand selection	Ligand filtering	Should be performed	Did not applied	No data
Ligands optimization	Ionization assessment	The ligands were ionized according to p*K_a_* and pH values of media	The research was performed without reference to p*K_a_* values of ligands and pH values of media	No data
	Generation of energetically possible conformations	Should be performed	Generation was performed without reference to potential energy calculation	No data
Target selection	Resolution of protein structure	Not more than 2.5 Å	More than 2.5 Å	No data
	Method of protein target structure obtaining	NMR spectroscopy	X-ray crystallography or cryogenic electron microscopy	No data
Target optimization	Control of histidine protonation	Should be performed	The structure of target did not reference biological conditions	No data
	Protonation of amino acids after X-ray crystallography or cryogenic electron microscopy	Should be performed	The structure of target did not reference biological conditions	No data
	Addition of missing residues and side chains after X-ray crystallography or cryogenic electron microscopy	Should be performed	Was performed without special tools	No data
	Addition of metals	Should be performed	The structure of target did not reference biological conditions	No data
Docking	Molecular docking software	Glide, GOLD	AutoDock, DOCK, FlexX	No data
Results assessment	Visual control	Should be performed	Structure defects were observed	No data
	Re-docking	Should be performed	The RMSD value is too high compared with the initial structure	No data
	Verification of docking results by in vitro study	Binding constant should be determined	The quantitative calculations were not performed	No data

**Table 3:** Comparison of the average affinity of flavonoid groups to target proteins in the AutoDock.

Flavonoid Group	Affinity to the Biological Target, kcal/mol *				
	ERα	E Protein DENV2-Thai	E Protein DENV2-My	Potassium Channel Kir6.1	Protein VacA
Flavones	−8.3 ± 0.6	−7.8 ± 1.3	−7.5 ± 0.9	−6.7 ± n/a	−8.5 ± 0.3
Flavonols	−7.9 ± 0.5	−8.4 ± n/a	−8.6 ± n/a	−8.1 ± n/a	-
Flavonol glycosides	-	−8.1 ± n/a	−7.7 ± n/a	-	-
Flavanones	−8.5 ± n/a	-	-	-	-
Flavanonols	−9.0 ± n/a	-	-	-	-

* A lower value of the scoring function corresponds to a better binding.

**Table 4:** Comparison of the average affinity of flavonoid groups to target proteins in Glide.

Flavonoid Group	Affinity to the Biological Target, kcal/mol *				
	ERα	Complex CA II-F	Arginase	Tec1	Rfg1
Flavones	−8.5 ± 0.3	−3.3 ± 0.0	-	-	-
Flavonols	−8.8 ± n/a	-	−8.1 ± n/a	-	-
Flavonol glycosides	-	-	−8.2 ± 0.3	-	-
Flavanones	−10.2 ± n/a	−2.7 ± 0.2	-	−7.7 ± n/a	−6.7 ± n/a
Flavanonols	-	−2.9 ± n/a	−8.2 ± n/a	−7.7 ± n/a	−4.9 ± n/a
Flavan-3-ols	-	−4.7 ± 0.6	-	-	-
Isoflavones	−9.0 ± 0.20	-	-	-	-
Dihydrochalcones	−8.3 ± n/a	-	-	-	-

* A lower value of the scoring function corresponds to a better binding.

## Data Availability

Data is contained within the article.
